# Bioaccumulation and Depletion of the Antibiotic Sulfadiazine ^14^C in Lambari (*Astyanax bimaculatus*)

**DOI:** 10.3390/ani13152464

**Published:** 2023-07-30

**Authors:** Patrícia Alexandre Evangelista, Felipe Machado de Oliveira Lourenço, Darmin Chakma, Chironjit Kumar Shaha, Almamy Konate, Rodrigo Floriano Pimpinato, Helder Louvandini, Valdemar Luiz Tornisielo

**Affiliations:** 1 Center for Nuclear Energy in Agriculture, University of Sao Paulo, Piracicaba 13416-000, Brazilhlouvandini@gmail.com (H.L.);; 2 Bangladesh Atomic Energy Commission, Dhaka 1349, Bangladesh; darmin.bmb@gmail.com (D.C.); chironjit39@gmail.com (C.K.S.); 3 Institute for the Environment and Agriculture Research (INERA), National Centre for Scientific & Technological Research (CNRST), Ouagadougou 04 BP 8645, Burkina Faso

**Keywords:** ecotoxicology, sulfonamides, human health risks, bioconcentration factor, fish

## Abstract

**Simple Summary:**

Antibiotics are present in the environment and can be bioaccumulated by organisms and transferred through the food chain, which poses a problem when considering fish meat consumption. The study focused on lambari, a fish with potential in aquaculture because of its ease of rearing in small-scale operations. The objective of the study was to measure the bioaccumulation and clearance of the antibiotic sulfadiazine (SDZ) in lambaris. The tests were divided into two phases: exposure and depletion. During exposure, the fish were fed medicated feed for seven days, and during depletion they were transferred to clean tanks and fed uncontaminated feed for another seven days. SDZ concentrations increased in the fish over the days, with the greatest accumulation occurring on day seven. After the depletion phase, SDZ concentrations decreased. The results showed that there was little bioaccumulation of SDZ in the fish, but that the compound was more present in the water. The results also indicated that the concentrations of SDZ were below the established maximum limit. This study contributes to the understanding of the dynamics of SDZ in an aquatic species native to Brazil.

**Abstract:**

Antibiotics are present in the environment, primarily due to their release through wastewater treatment plants, agricultural practices, and improper disposal of unused medications. In the environment, these drugs can be bioaccumulated by organisms and transferred along the food chain. This is a problem when considering the consumption of fish meat. In the United States, legislation stipulates that the maximum residue limit for sulfadiazine (SDZ) should not exceed 100 μg kg^−1^. Lambari fishes have potential economic importance in aquaculture, as they are relatively easy to breed and can be raised in small-scale operations. Finally, studying the biology and ecology of lambari could provide valuable information about freshwater ecosystems and their inhabitants. The current work aimed to measure the bioaccumulation and depletion of the antibiotic SDZ ^14^C in lambari (*Astyanax bimaculatus*). For this purpose, the tests were divided into two stages; seven days of exposure and seven days of depletion, where one fish was randomly selected and sampled every day. In the exposure phase, the fish were fed the medicated feed three times a day at a concentration of 2.5 mg·g^−1^. The control fish were fed uncontaminated feed. For the depletion phase, the remaining lambari were transferred to clean tanks and fed uncontaminated feed three times a day. The fish samples were burned in the Oxidizer and the reading of radioactivity was performed in a liquid scintillation spectrometer. It is worth noting that on day 7 and day 14, the water in the aquariums was filtered through filter paper to collect the metabolic excrement. SDZ concentrations increased over the days and accumulation occurred in the fish, with day seven presenting the maximum accumulation value of 91.7 ng·g^−1^ due to feeding uptake. After the depletion phase on day 13, the value found was 0.83 ng·g^−1^. The bioconcentration factor calculated was 20 L·kg^−1^. After the bioaccumulation period, the concentrations of SDZ in the water and excreta were 4.5 µg·L^−1^ and 363.5 ng·g^−1^, respectively. In the depletion period, the concentrations in the water and excreta were 0.01 µg·L^−1^ and 5.96 ng·g^−1^, respectively. These results imply that there was little SDZ bioaccumulation in the fish, but that it was distributed in larger amounts in the water. This is due to the physicochemical properties of the molecule with the low Log P value. Regarding the maximum residue limit, the value was below the established value. This study contributes to understanding SDZ dynamics in an aquatic species native to Brazil.

## 1. Introduction

Drugs can present a high risk to the environment because they are designed to have a biological effect even at low doses [1]. Among the most widely used drugs are antibiotics, which are natural, synthetic, and/or semi-synthetic compounds containing antimicrobial activities that are widely used to treat diseases caused by bacterial infections in humans, plants, and animals [2,3].

Global consumption of antibiotics is estimated to exceed 63,151 tons per year [2]. That said, environmental pollution caused by antibiotics has become a major problem today because, besides their extensive use, there is undue discharge of these contaminants into the environment, either via wastewater treatment plants (WWTPs) or waste disposal from agricultural activity [4].

Although human consumption has received the most attention, antibiotics are employed on a large scale in the livestock, aquaculture, pig, and poultry farming sectors. This generates considerable concern and relevance because widespread administration of these drugs can lead to the development of resistant bacterial pathogens [4,5,6]. 

In aquaculture, antibiotics are often administered orally via the feed, as this is the most economical method to treat large numbers of fish [7]. The aquaculture industry is a major consumer of antibiotics, as the expanding population and consequently, high food consumption, has led to increasing incorporation of these substances in the treatment of diseases that may affect fish, as well as to maximize yields [8]. 

SDZ is classified as a sulfonamide, a large group of synthetic antibacterial compounds. In their mechanism of action, antibiotics of this class inhibit the conversion of p-aminobenzoic acid, ceasing the synthesis of folic acid, purine, and DNA. Resistance has been reported in microorganisms previously susceptible to sulfonamides [9]. SDZ is a widely used antibiotic in aquaculture and has been detected in significant concentrations in freshwater environments [9,10,11]. In addition, despite the known use of SDZ in fish farming, its use is not regulated and there is no maximum residue limit (MRL) determination in Brazilian legislation [12]. In the United States and the European Union, the MRL for SDZ is 100 μg kg^−1^ (equivalent to 1.0 10^−4^ mg·g^−1^ or 100 ng·g^−1^) [13].

Bioaccumulation of this compound in fish may represent a risk to human health and the conservation of aquatic biodiversity. Thus, the increased use of antibiotics in animal production has led to global apprehension about the effects of these compounds on aquatic ecosystems, as aquaculture wastewater has become an important source of antibiotics for freshwater environments.

Lambari fish farming is a growing market in Brazil, with production of around 1000 tons per year, cultivated by small producers [14]. Besides the breeding of the species, wild lambari are widely consumed by communities living near freshwater bodies [15]. Lambari have the most diverse feeding habits, consuming resources of allochthonous origin (fruits, seeds, and terrestrial insects) and autochthonous origin (insects and aquatic plants, scales, and oocytes, among others). They explore all trophic levels, that is, they can be primary (feeding on plants and phytoplankton), secondary, or tertiary consumers (ingesting zooplankton, insects, and fish). Sediments and debris can also be consumed by this species [16]. These characteristics contribute to their ingestion/exposure to antibiotics. Thus, it is relevant to evaluate the bioaccumulation and depletion of SDZ in lambari, for better understanding of the environmental fate of this pharmaceutical product.

The current study aimed to perform the bioaccumulation and depletion of ^14^C-SDZ in lambari (*Astyanax bimaculatus*). The results of this research could assist in the development of public policies for the management of antibiotics in animal production and the conservation of aquatic ecosystems and provide relevant data about the environmental and public health effects of SDZ use in aquaculture farms.

## 2. Materials and Methods

### 2.1. Test Substance

The sulfadiazine (4-Amino-N-(2-pyrimidinyl)benzenesulfonamide, N1-(Pyrimidin-2-yl)sulfanilamide—C10H10N4O2S) used in the experiment was purchased from Sigma-Aldrich (Hen-5154x Riedel). The radiochemical purity ^14^ of the C SDZ was >99% (ascertained), the specific activity was 3.58 MBq.mg^−1^, and the activity was 9.25 MBq (Table 1).

### 2.2. Incorporation of SDZ in the Feed

The feed used in the experiment was purchased commercially and contained 45% protein (GuabiTech, Campinas, SP, Brazil). The amount of ^14^C SDZ (Sigma-Aldrich; Saint Louis, MO, USA, Hen- 5154X Riedel) used in the experiment was the one that gave the lowest reading signal in the liquid scintillation spectrometer. Thus, the amount of feed per fish was the equivalent of 1% of body weight, 12 mg·day^−1^, totaling 3528 mg of feed contaminated with 5540.38 Bq of radioactive product. The feed was homogenized after the application of the ^14^C SDZ to ensure that all of the drug was evenly distributed in the feed. For confirmation, seven 50 mg samples of contaminated feed were used for the liquid scintillation spectrometer reading.

### 2.3. Experimental Conditions

The experiment was previously approved by the Ethics Committee on Animal Use of the Center for Nuclear Energy in Agriculture, Piracicaba SP, Brazil (Protocol number 0007/2022).

The bioaccumulation and depletion study using lambari was conducted to evaluate how the elimination of the SDZ molecule works after treatment. The experiment was performed based on the guidelines of the Organization for Economic Cooperation and Development standard no. 305 [17] and VICH-FDA [18], with adaptations for the use of radiolabeled molecules.

The lambari fingerlings were acquired from a commercial fish farm, without distinction between male and female. A total of 100 healthy fish were placed in a tank with 200 L of (chlorine-free) water. The acclimatization took place over 15 days, during which the fish were fed three times daily with commercial fish food. The water temperature was maintained at 25 °C ± 1 °C and pH 7. Feeding of the fry was stopped 24 h before the experiment.

The tests were divided into two stages, seven days of exposure and seven days of depuration. After the acclimatization process, 84 fish (approximately 5 cm long and weighing approximately 1.0 to 1.4 g) were used in the experiment. The experiment was conducted in 8 L stainless steel tanks with three replicates and two controls, with 14 fish placed in each tank. The water temperature was maintained at 25 °C ± 1 °C, pH 7, under permanent aeration, with a 12 h photoperiod. The water temperature was maintained at 25 °C ± 1 °C, pH 7, under permanent aeration, with a 12 h photoperiod. Water temperature and pH were analyzed daily. As the room is climate-controlled, the temperature remained stable. Regarding pH, the variation was not significant (<±0.5) until the switch to depletion phase. The aquaria had constant aeration which kept the dissolved oxygen level stable.

The exposure phase of the fish to the antibiotic lasted 7 consecutive days, during which the medicated feed was offered three times a day. Control fish were fed uncontaminated feed. For the depletion phase, the remaining lambaris were transferred to clean tanks with 4 L of water and fed uncontaminated feed three times a day. It is worth noting that one fish was randomly selected and sampled during the exposure (days 1, 2, 3, 4, 5, 6, and 7) and depletion (8, 9, 10, 11, 12, 13, and 14) periods of each tank; the selected lambari was euthanized with a 0.2 mg.mL^−1^ benzocaine immersion bath and frozen for later analysis.

### 2.4. Antibiotic Residues Analyses

Fish samples were burned in the Oxidizer OX500 (RJ—Harvey Instrument Corporation, Buffalo, NY, USA) in an oxygen-rich atmosphere, where 10 mL of scintillation liquid (permafluor) was used per sample. Measurement was by liquid scintillation counting (LSC) with a Tri-Carb 2910 TR LSA counter (Perkin Elmer, Waltham, MA, USA).

On day 7 (the last day of bioaccumulation) and day 14 (the last day of depletion), the aquarium water was filtered through filter paper (Whatman 42) to collect background material (metabolic excrement). The analyses of the excreta were conducted in the same way as the procedures described for fish. In addition, water samples from each aquarium were sampled in triplicate at the exposure phase (beginning and end) to determine the radiolabeled molecule, using liquid scintillation counters (LSC) with a Tri-Carb 2910 TR LSA counter (Perkin Elmer) [19].

### 2.5. Data Treatment

The data from the bioconcentration phase were fitted to an exponential growth model (1) and from the elimination phase to a one-phase exponential decay model (2) to determine the uptake and elimination coefficients and the elimination rate of SDZ in lambari [20]. For this purpose, GraphPad Prism software, version 9.5.1 for Windows, San Diego, CA, USA, was used.

(1) $$C_{t}=A\left(e^{-k_{e}t}\right)$$

(2) $$C_{t}=\frac{KuCw}{Ke}\left(1-e^{-k_{e}t}\right)$$

where C_t_ is the concentration of SDZ in the fish at time t (ng·g^−1^), A is the pre-exponential term representing the initial concentration of SDZ (ng·g^−1^), Ke is the elimination rate constant, Ku is the uptake rate constant, Cw is the concentration in the water (ng·L^−1^), and t is the elapsed time (d).

In turn, the half-life time was determined by the following Equation (3).

(3) $$t_{1/2}=\frac{\ln2}{Ke}$$

Finally, the Bioaccumulation Factor (BCF) was determined from Equation (4).

(4) $$BCF=\frac{Ca}{Cw}$$

where Ca is the concentration in animals (ng·g^−1^) and Cw is the concentration in water (ng·L^−1^).

### 2.6. Radioactive Waste

All radioactive materials produced in the experiment were stored in suitable containers. The receipt, storage, and control of radioactive material were recorded on forms prepared for this use. All these steps are under the responsibility of the CENA/USP Radiological Protection Service, obeying the determinations of the National Nuclear Energy Commission (NORM CNEN-NE-6.05).

## 3. Results

**Figure 1:**
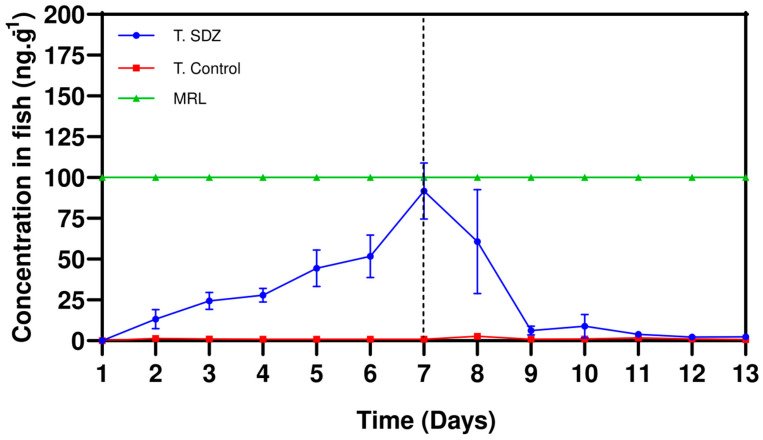
Concentration as a function of time of bioaccumulation (1–7 days) and depletion (7–13 days) of ^14^C-sulfadiazine offered in the diet of lambari (*Astyanax bimaculatus*). Symbols represent standard deviation (*n* = 3). T. SDZ = Sulfadiazine treatment; T. Control = Control treatment; MRL = Maximum Residue Limit.

The results of the experiment and the mass balance showed that 0.21% of the SDZ applied in the diet bioaccumulated in the fish, while the remainder remained in the excreta (0.69%) and a large part in the water (115%). The bioaccumulation (1–7 days) and depletion (7–13 days) profiles of lambari (*Astyanax bimaculatus*) to the diet with the SDZ (2.54 10^6^ ng·g^−1^) are displayed in Figure 1.

The results showed that during the exposure phase, the concentration of SDZ increased in the fish tissues over time, reaching a peak on day 7 at a concentration of 91.72 ng·g^−1^. After two days of the depletion phase (7–9 days) the organisms had eliminated 93.12% of the accumulated SDZ, and at the end of the experiment the concentration was 0.83 ng·g^−1^. It was observed that during the exposure and elimination phase, the concentration was below the MRLs (100 ng·g^−1^).

Table 2 displays the toxicokinetic parameters determined in the bioconcentration and depletion phases of SDZ in lambari, which include the velocity constants (ke and ku), half-life time (T_1/2_), and bioconcentration factor (BCF).

At the end of the exposure phase, the concentration of SDZ in the water was 4.6 × 10^−3^ mg·L^−1^, whereas, in the depletion phase, the concentration was 2.5 × 10^−5^ mg·L^−1^. In turn, in the excreta, the concentrations of SDZ in the exposure and elimination phases were 3.6 × 10^−4^ and 5.9 × 10^−6^ mg·g^−1^, respectively (Table 3).

On day 12 of the experiment (not counting day 0), deaths occurred in the aquariums and the experiment was terminated. This fact is not linked to the use of the substance analyzed here. It is important to point out that although the experiment was terminated ahead of schedule, this did not compromise the results of the analyses.

## 4. Discussion

The data obtained demonstrate that ^14^C-SDZ has a low potential for bioaccumulation in the studied species. This may be related to the physicochemical properties of the molecule, as well as the physical characteristics of the organism used in the analysis. 

SDZ is an antibiotic that is frequently detected in freshwater environments in several regions of the world [21,22,23,24,25]. In the current study, it was noted that this compound demonstrated a higher concentration of distribution in the water, because it is highly soluble. However, its presence in the aquatic environment can bring numerous risks, such as bacterial resistance [26,27].

SDZ is highly soluble in water (hydrophilic), which can be explained by its physicochemical properties, especially its octanol-water partition coefficient (Log P) with a value equal to −0.09 [28]. This may have contributed to higher amounts of the ^14^C-SDZ remaining in the water. Lipophilic organic compounds, with high Log *p* values (>3), tend to bioaccumulate in lipid tissues. In turn, hydrophilic compounds with low Log *p* values (<3) are more soluble in water and thus are not attracted to lipid materials and tend to bioaccumulate to a lower extent [29].

Furthermore, the dynamics of ^14^C-SDZ may also have been affected by the pH and temperature of the aquarium. The studies were carried out under conditions of room temperature (25 °C) and pH = 7 (neutral), which are favorable for the solubility of SDZ in water. pH can interfere with the ability of SDZ to bioaccumulate by influencing the ionization of the molecule, which can interfere with its solubility in water. Higher pH values (>7) tend to decrease the BCF of SDZ in organisms, which is due to the higher non-ionized fraction of the molecule [30]. At neutral pH, as in aquariums, SDZ is predominantly in its ionized form due to its acid–base dissociation constant (pKa) of 6.5, which contributes to the decreased permeability of the molecule in fish cell membranes. 

Room temperature is seen as optimal for lambari metabolism, which may have contributed to the low bioaccumulation of the molecule in fish and the rapid depletion of the molecule. At different temperatures (14.0 and 19.5 °C), depletion of the SDZ occurred more quickly at the higher temperature [31]. During the exposure phase (1–7 days) an increase in the concentration of ^14^C-SDZ in the tissues of the organisms was noted, reaching its peak on day 7, at a concentration of 91.72 ng·g^−1^. This shows that the fish absorbed the ^14^C-SDZ supplied in the diet and water. However, in the depletion phase period (7–13 days), 93.12% of the accumulated ^14^C-SDZ was eliminated by the fish, indicating that this substance is rapidly excreted by the kidneys.

Regarding the absorption and elimination of the substance, our results are in agreement with some studies, but differ from others. This is because in some species SDZ is rapidly absorbed and rapidly eliminated [31,32], whereas in others the opposite occurs [33,34]. Studies suggest that the absorption of SDZ may be decreased by the metabolism of the organism studied [33,34].

Lambari have an accelerated metabolism and are small fish (up to 10 cm in length), characteristics that restrict their ability to bioaccumulate lipophilic molecules such as SDZ. Furthermore, the T_1/2_ of SDZ is short, showing a value equal to 1.13 d, i.e., upon ingestion it is rapidly eliminated by the fish. Fish metabolism can be considered an important factor in the depletion of SDZ in fish tissues through biotransformation and excretion processes [35]. However, the rate and efficiency of this process can differ between fish species and be influenced by dietary and environmental conditions.

Zhao et al. [35], evaluated the bioaccumulation of different concentrations of SDZ in common carp (*Cyprinus carpio*) and showed that the concentration of the substance increased with exposure time, reaching a peak on day 24. During the depletion phase, the concentration of the substance decreased significantly until day 24 (8 days of depletion). The authors observed that bioaccumulation of SDZ in fish tissues is dependent on exposure concentrations. According to studies by Hendriks et al. [36], moderately hydrophobic substances had low absorption rates and high elimination rate constants, while the reverse occurred for very hydrophobic substances.

In the study by Vilca et al. [19], using rainbow trout (*Oncorhynchus mykiss*) with SDZ fed in the diet, the highest accumulation of the substance in the organisms occurred on day 4, reaching 5.10 × 10 ^−4^ mg·g^−1^. However, on the seventh day of depletion, the substance was significantly eliminated by the organisms, reaching 1.0 × 10^−4^ mg·g ^−1^. The authors concluded that these organisms should not be consumed, since the concentration of SDZ used (0.0330 μg·mg^−1^) puts human health at risk because, after the depletion phase, the concentrations exceeded the MRL (100 ug·kg^−1^).

In a study by Silveira et al. [37], with *Danio rerio,* the SDZ showed low bioconcentration in organisms, in which the bioaccumulated amount of SDZ (4.15 and 5.41 Bq) was proportionally eliminated (3.79 and 5.21 Bq).

The results obtained in the current study demonstrate that bioaccumulation and depletion of SDZ in organisms is dependent on concentration, exposure time, and environmental conditions of exposure, such as pH and temperature. The rate of accumulation of organic compounds in organisms is dependent on the exposure time and the concentration of the substance. 

BCF is a parameter that demonstrates the ability of a chemical compound to accumulate in living organisms relative to its concentration in the environment. The calculated value for BCF was 20 L·kg^−1^ of SDZ in lambaris. This shows that SDZ has a low capacity to bioaccumulate in lambari tissues and is readily eliminated. The BCF found indicates that the concentration of SDZ in fish is close to 1/50 (or 2%) of the concentration in their aquatic environment. BCFs below 1000 L·kg^−1^ were observed for most antibiotics [32]. 

Zhu et al. [32], found BCF values after SDZ exposure in sea cucumbers (*Apostichopus japonicus*) of between 2.1–891.5 (L·kg^−1^). Zhao et al. [35] found BCFs in the range of 2.73–165.73 L·kg^−1^ for SDZ in common carp (*Cyprinus carpio*). Vilca et al. [19], found BCF of 4100 (L·kg^−1^) in rainbow trout (*Oncorhynchus mykiss*).

It is worth noting that BCF values can change as a result of various aspects, such as fish size, species, age, metabolism, exposure time, and different environmental conditions. Furthermore, the physicochemical properties of SDZ—solubility, lipophilicity, and rate of metabolic elimination—are considered to be factors that influence BCF values.

In addition, it was evidenced that the presence of ^14^C-SDZ in the lambari does not present a risk to human health, since the concentration of the substance in the organisms during the bioaccumulation and depletion phases was below the established MRL (100 ng·g^−1^). However, it is important to note that the indiscriminate use of antibiotics in aquaculture can lead to the development of bacterial resistance.

## 5. Conclusions

The results of this research demonstrated that SDZ shows low bioaccumulation in lambari (*Astyanax bimaculatus*) and high depletion, remaining at higher concentrations in the water and sediment. The concentration bioaccumulated by organisms does not present a risk to human health. Physicochemical characteristics such as low liposolubility substantiated these results.

## Figures and Tables

**Table 1:** Physical-chemical characteristics of Sulfadiazine.

Substance	Chemical Structure	Molecular Weight	Log P	Solubility in Water
Sulfadiazine (CAS: 68–35–9)	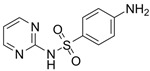	250.278 g·mol^−1^	−0.09	77 mg·L^−1^

**Table 2:** Toxicokinetic parameters and bioconcentration factor of ^14^C-sulfadiazine offered in lambari diets (*Astyanax bimaculatus*).

Parameters	Bioaccumulation	Depletion
ke (d^−1^)	0.3956	-
r^2^	0.7607	-
BCF (L·kg^−1^)	20	
A (ng·g^−1^)		186.2
ku (mL g^−1^d^−1^)		0.6155
T_1/2_ (d)		1.126
r^2^		0.609

ke: elimination constant (d^−1^), r^2^: model fit coefficient; A: pre-exponential term; ku: absorption constant (L kg^−1^d^−1^), T_1/2_: half-life time.

**Table 3:** Concentration of ^14^C-SDZ in water and sediment after exposure (1–7 days) and elimination (7–13 days) steps offered in the lambari diet (*Astyanax bimaculatus*).

Water (mg·L^−1^)			Sediment (mg·g^−1^)	
	SDZ	Control	SDZ	Control
Bioaccumulation	4.6 × 10^−3^	4.5 × 10^−6^	3.6 × 10^−4^	4.8 × 10^−6^
Depletion	2.5 × 10^−5^	0.5 × 10^−6^	5.9 × 10^−6^	1.5 × 10^−6^

## Data Availability

Not applicable.
